# Supplementary material for: Colloidal synthesis of large near-bulk InAs quantum dots through seeded and seedless growth using cluster precursors
Source: Nat Commun. 2026 Feb 12;17:1700. doi: 10.1038/s41467-026-69409-w (PMC12909979; doi:10.1038/s41467-026-69409-w)
Supplement: Supplementary file 1 — Supplementary Information [file 41467_2026_69409_MOESM1_ESM.pdf]

SUPPLEMENTARY INFORMATION

Colloidal synthesis of large near-bulk InAs quantum dots through seeded and seedless growth using cluster precursors

Ekaterina Salikhova[1,2,*], Alf Mews[2], Hendrik Schlicke[1,3], Jan Steffen Niehaus[1,*]

[1] Fraunhofer IAP-CAN, Department Quantum Materials, Grindelallee 117, 20146 Hamburg, Germany

[2] University of Hamburg, Physical Chemistry Department, Grindelallee 117, 20146 Hamburg, Germany

[3] Present address: Leibniz Institute of Polymer Research Dresden, Hohe Straße 6, 01069 Dresden, Germany

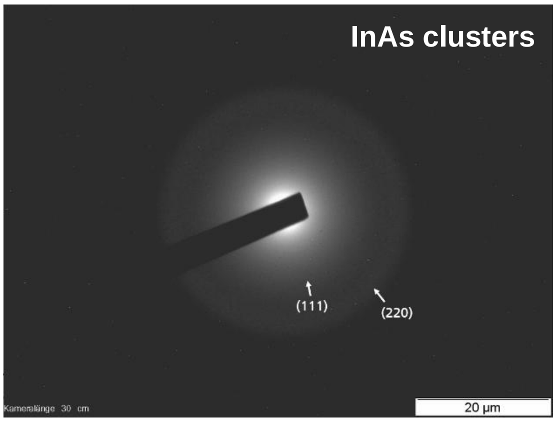

Supplementary Fig. 1: SAED pattern of purified InAs clusters with an absorption maximum at 556 nm.[1,2]

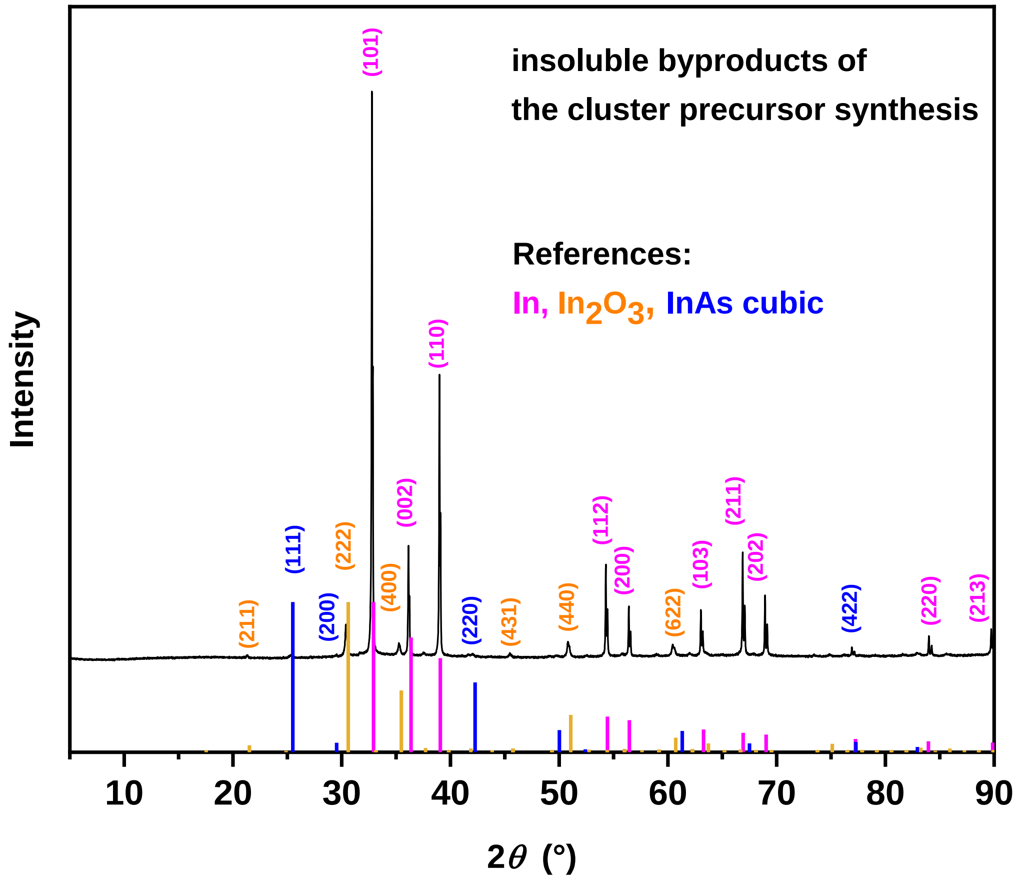

Supplementary Fig. 2: XRD diffractogram of insoluble byproducts of a cluster synthesis conducted at 130 °C for 76 min.[2] Source data are provided as a Source Data file.

Supplementary Table 1: Overview of the hot injection synthesis parameters for InAs clusters and their optical properties, characterized after removing insoluble byproducts. The synthesis marked with asterisk resulted in slightly larger clusters due to a brief temperature overshoot at the beginning of the reaction.[1,2]

temperature	reaction time	1st absorption peak of InAs clusters
110 °C	60 min	487 nm
110 °C	200 min	532 nm
120 °C	30 min	478 nm
130 °C	30 min	497 nm
130 °C	60 min	514 nm
130 °C	76 min	526 nm
130 °C*	60 min	556 nm
130 °C	90 min	562 nm
180 °C	2 min	607 nm
180 °C	15 min	663 nm

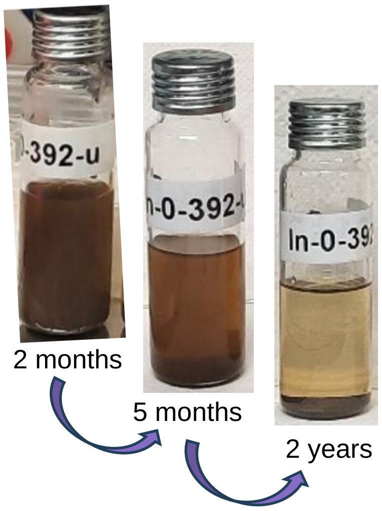

Supplementary Fig. 3: Photographs of a sample stored at room temperature under nitrogen conditions at three different time points. The sample was prepared at 130 °C following the cluster synthesis protocol described in the Methods section, but the reaction was quenched early, after 16 min, and then allowed to proceed at RT for an extended period. The brown and turbid supernatant gradually became clear during two years of storage without centrifugation or shaking.[1,2]

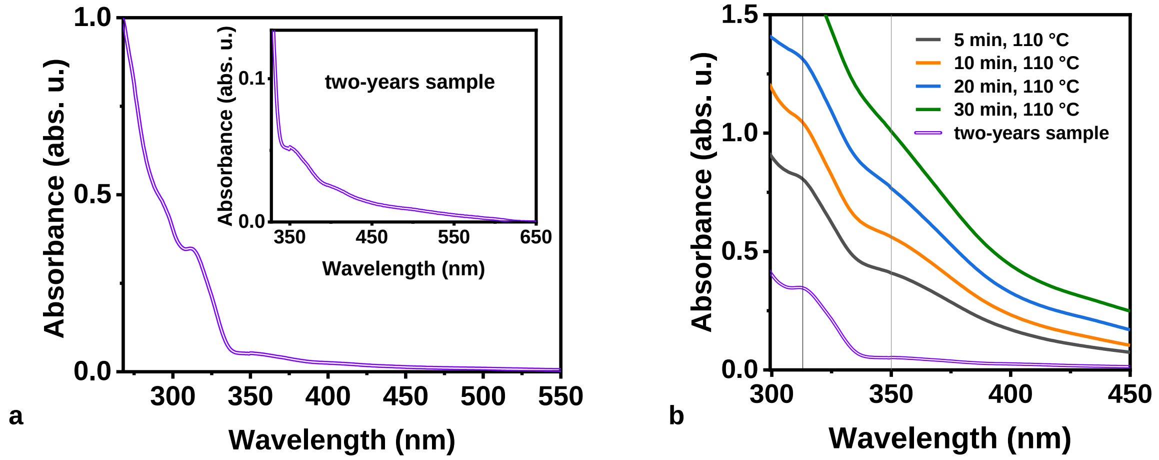

Supplementary Fig. 4: Absorption spectrum of the structures from the 2-years experiment corresponding to Suppl. Fig. 3.[1,2] Source data are provided as a Source Data file. **a** Absorption spectrum, including a zoomed-in inset, showing UV-active structures with multiple absorption maxima. **b** The same spectrum in comparison to absorption spectra of the early-stage aliquots shown in Fig. 3c. Absorption maxima are marked by two grey lines parallel to *y*-axis.

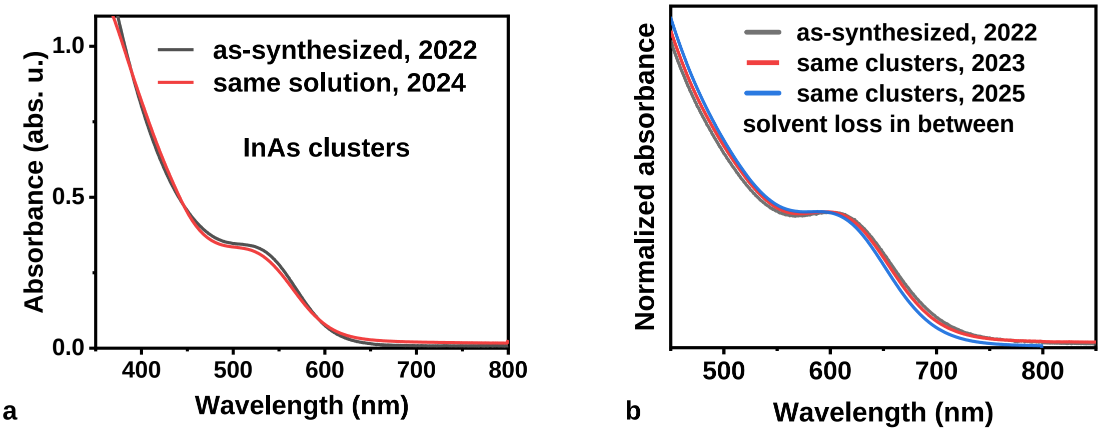

Supplementary Fig. 5: Absorption spectra demonstrate stability of InAs clusters over time. The samples were stored at room temperature under nitrogen conditions.[1,2] Source data are provided as a Source Data file. **a** InAs cluster solution measured twice with a two-year interval; insoluble byproducts were separated before the measurements. Spectra were not normalized. **b** Purified cluster sample measured in toluene three times within three years, with solvent loss in between. Spectra were normalized to their respective absorption maxima.

Supplementary Note 1: The feasibility of synthesizing stable clusters via a RT reaction was investigated, following the standard cluster synthesis protocol. The reaction mixture color shifted from yellow to orange, red, and pink-red within 33 minutes, darkening after 18 h (Suppl. Fig. 6a-j). After an additional 73 h (Suppl. Fig. 6k), the supernatant lightened, and a greater amount of metallic byproduct was visible. After two weeks, the supernatant turned light yellow (Suppl. Fig. 6m). The absorption spectrum of the pink-red sample showed sharp peaks (Suppl. Fig. 7), suggesting the formation of metallic NPs. The plasmonic nature of these structures was further investigated in a separate experiment (Suppl. Fig. 8), where they exhibited aggregation, scattering, and broadening of absorption maxima in the non-solvent ethanol. This behavior is typical for metallic nanoparticles due to localized surface plasmon resonance (LSPR).[3]

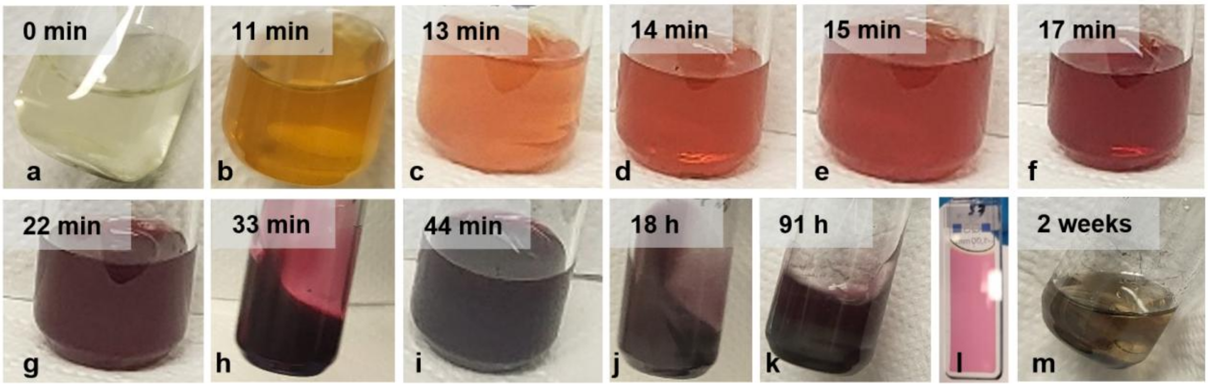

Supplementary Fig. 6: Photographs of the reaction mixture during the RT synthesis, depending on the reaction time.[1,2] **a-k** Mixture during the first 91 h. **l** Supernatant of the 18 h sample in a cuvette after the separation of insoluble byproducts. A yellowish hue appeared at the meniscus, caused by the sedimentation of pink-colored structures. **m** Reaction mixture after 2 weeks.

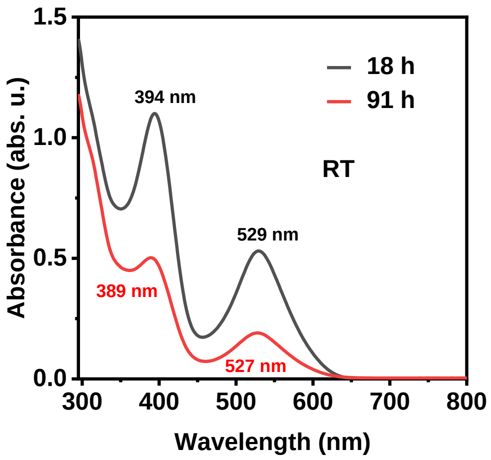

Supplementary Fig. 7: Absorption spectra of the RT reaction mixture after 18 h (black line) and 91 h (red line), corresponding to Suppl. Fig. 6j,k, showing a decreasing nanoparticle concentration and a slight blueshift over time. Before measurements, insoluble byproducts were removed via centrifugation.[1,2] Source data are provided as a Source Data file.

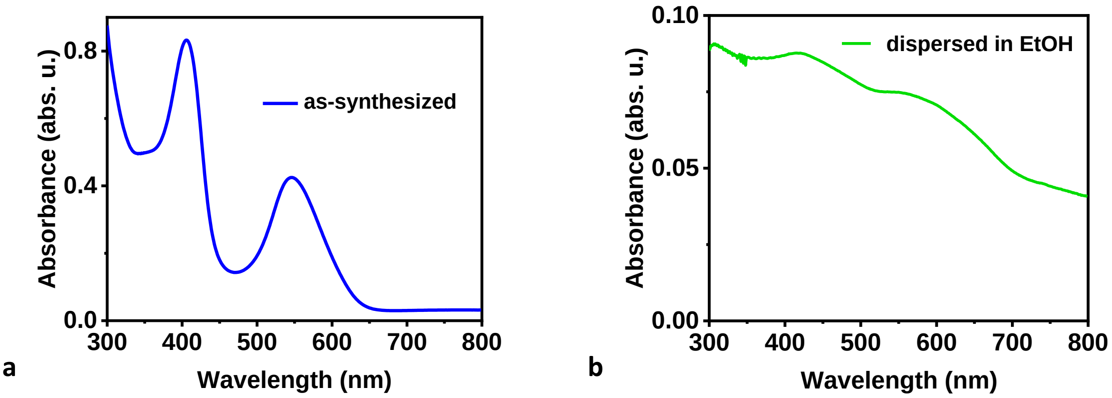

Supplementary Fig. 8: Absorption spectra of the RT reaction mixture, measured in different media.[2] Source data are provided as a Source Data file. **a** As-received sample after removing insoluble byproducts. **b** The same particles, after precipitation and dispersion in ethanol.

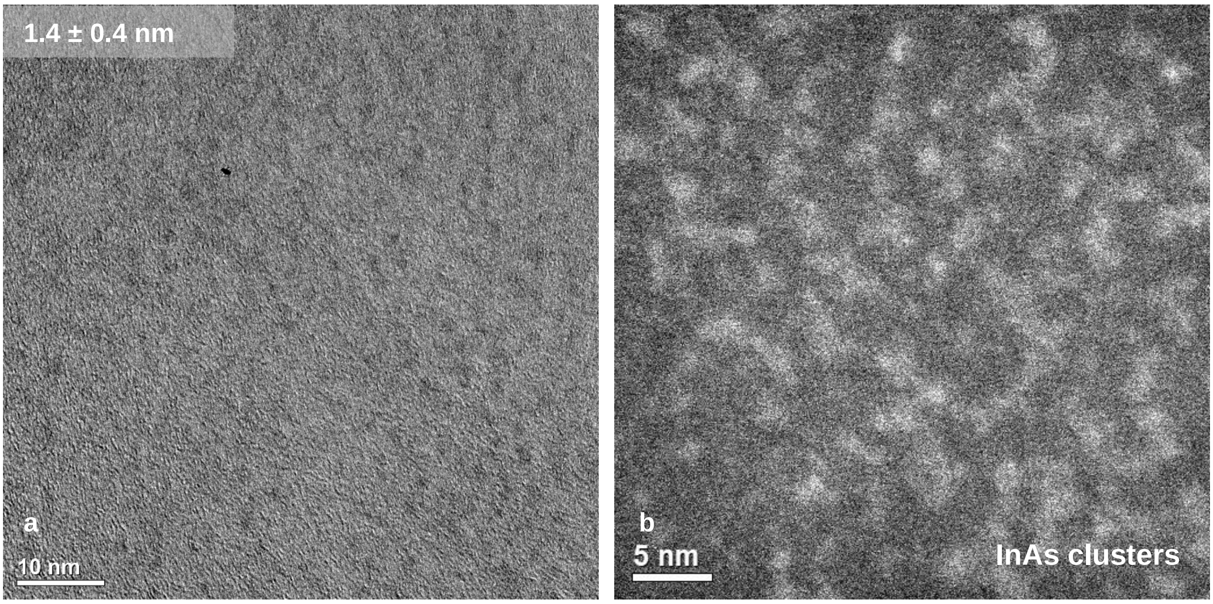

Supplementary Fig. 9: InAs clusters synthesized at 130 °C for 1 h, corresponding to Fig. 4a. Cluster width was taken into account for size determination.[2] **a** HR-TEM image and **b** HAADF-STEM image of clusters.

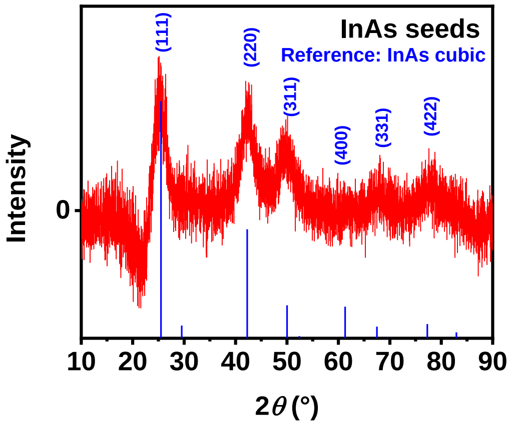

Supplementary Fig. 10: XRD diffractogram of small monodisperse InAs QDs synthesized via standard heat-up approach, later used as seeds for the seeded growth synthesis.[1,2] The sample was covered with Kapton® polyimide tape, and the background of an empty, tape-covered wafer was subtracted from the measured data. Source data are provided as a Source Data file.

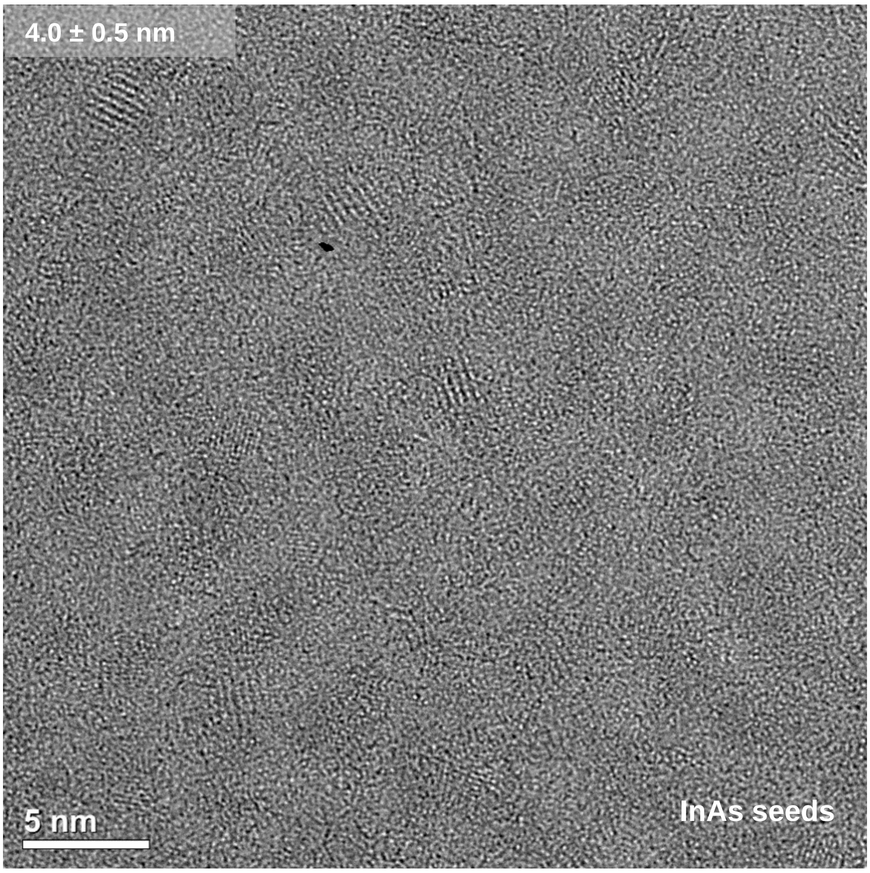

Supplementary Fig. 11: HR-TEM image of small monodisperse InAs QDs, later used as seeds for the seeded growth synthesis, displaying lattice fringes and high crystallinity.[1,2]

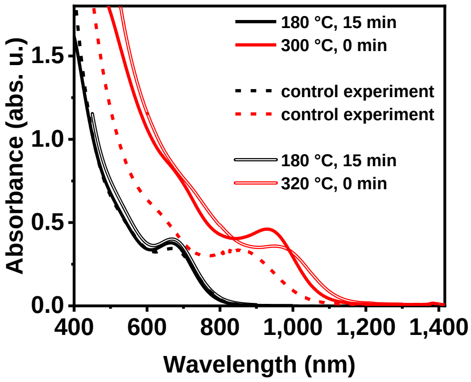

Supplementary Fig. 12: Absorption spectra of different InAs samples from three experiments showing InAs clusters (black lines) synthesized at 180 °C for 15 min and the resulting small InAs QDs (red lines) after heating the clusters to target temperatures. Single lines represent the standard heat-up method from Fig. 4e-g, while dashed lines illustrate a control experiment where insoluble byproducts were removed before heating to 300 °C. Double lines correspond to the 320 °C control experiment. The absorption spectra were taken after separation of insoluble byproducts.[1,2] Source data are provided as a Source Data file.

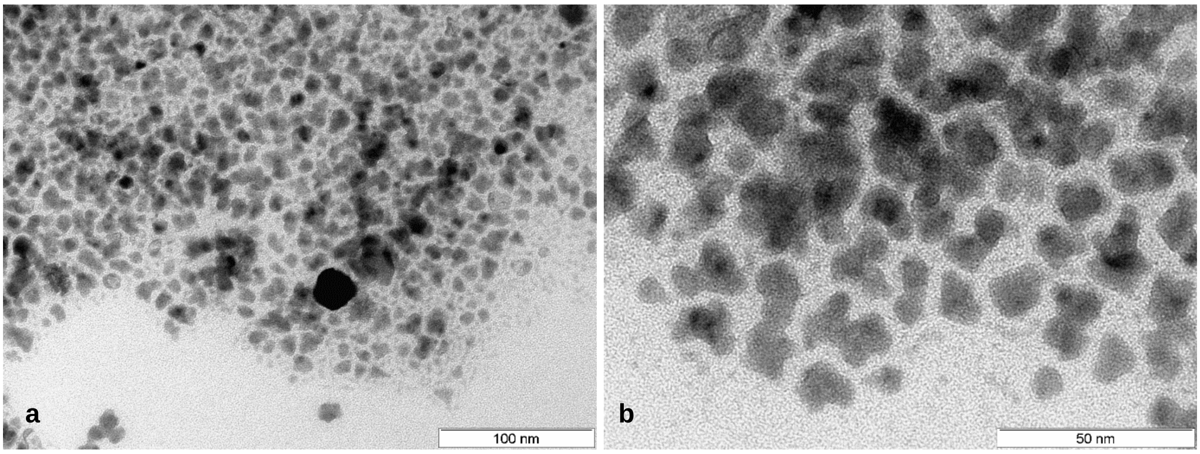

Supplementary Fig. 13: Insoluble byproducts of the standard heat-up approach to synthesize InAs seeds. **a,b** Representative TEM images.[2]

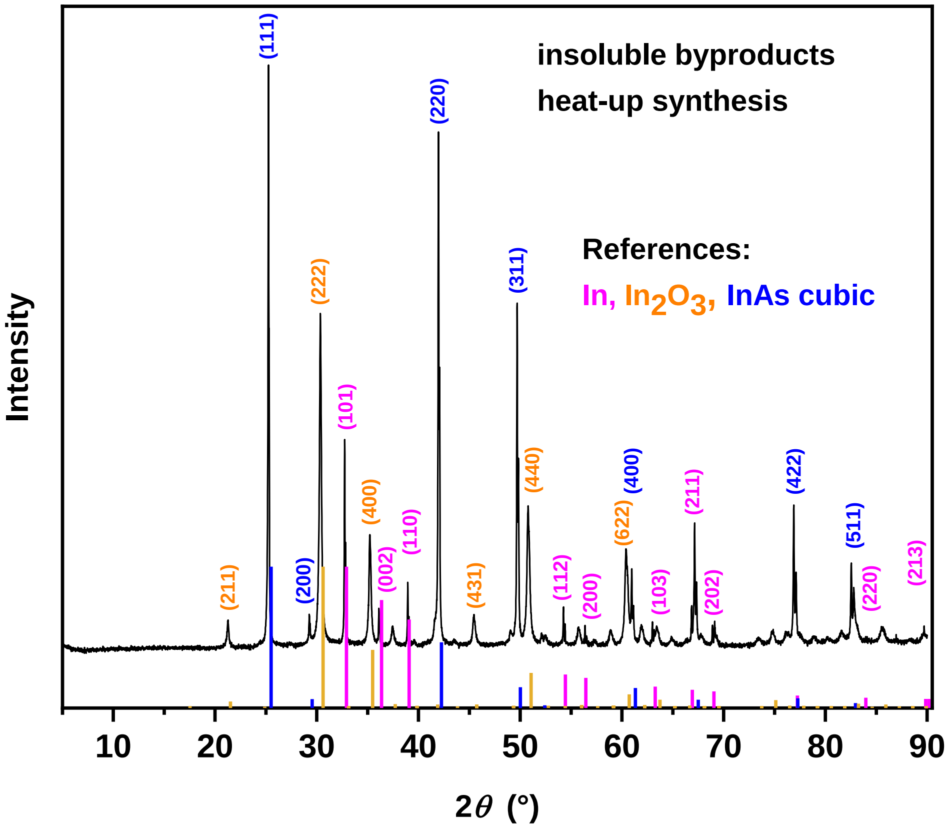

Supplementary Fig. 14: XRD diffractogram of insoluble byproducts of the standard heat-up approach to synthesize InAs seeds.[2] Source data are provided as a Source Data file.

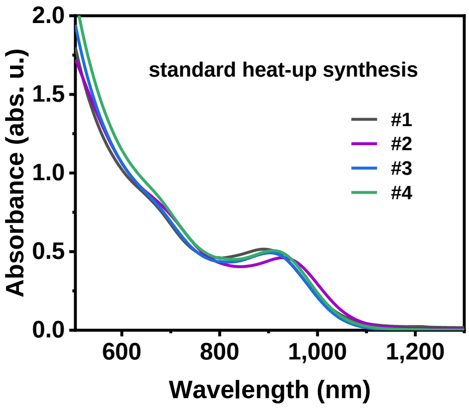

Supplementary Fig. 15: Absorption measurements of four seed solutions, obtained from four independently repeated standard heat-up experiments, measured each after removing insoluble byproducts via centrifugation, without further purification.[2] Source data are provided as a Source Data file.

Supplementary Table 2: Results of the elemental analysis on InAs QDs synthesized via the standard heat-up approach. The analysis was conducted three times.

analysis #	indium (In)	arsenic (As)
1	1,026 mg L^{-1}	585 mg L^{-1}
2	1,037 mg L^{-1}	576 mg L^{-1}
3	1,038 mg L^{-1}	577 mg L^{-1}

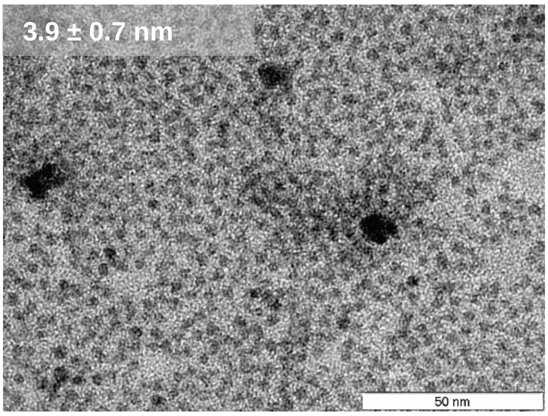

Supplementary Fig. 16: TEM image of NPs synthesized in a control heat-up experiment, where insoluble byproducts were removed prior to heating to 300 °C (see also Suppl. Fig. 12, red dashed line). InAs QDs and larger, darker appearing NPs are present.[1,2]

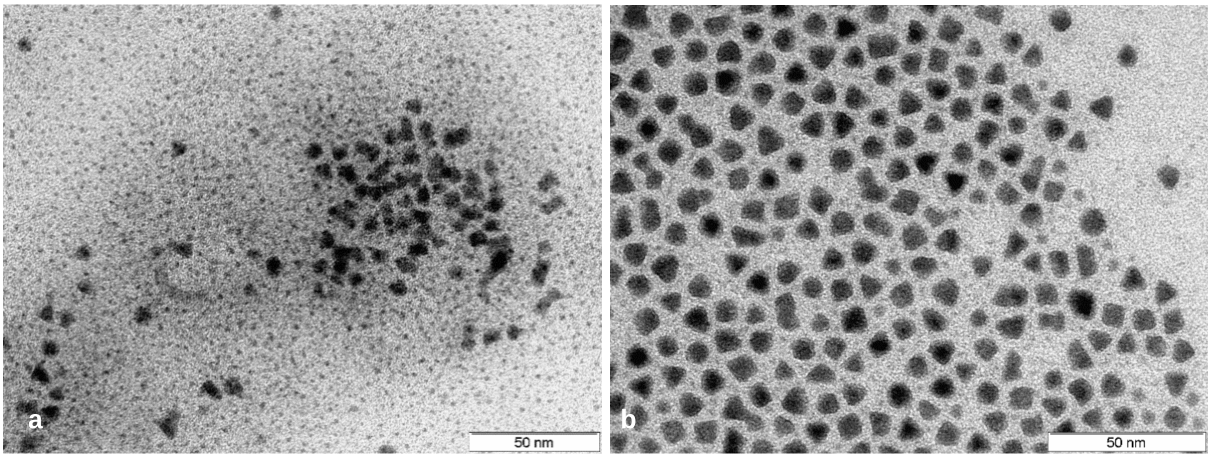

Supplementary Fig. 17: TEM images of InAs NPs resulting from unoptimized seeded growth parameters in the representative synthesis #2 outlined in Suppl. Table 3. **a,b** TEM images showing secondary nucleation evidenced by the appearance of NPs much smaller than the initial seeds, along with aggregated particles due to interparticle ripening.[2]

Supplementary Table 3: Overview of the unoptimized reaction parameters for the seeded growth syntheses conducted at 300 °C, where agglomerated NPs were observed in the TEM images.[2] The table lists the wavelength of the first absorption (abs.) maximum *λ,* optical density OD, and volume *V* of cluster and seed solutions, injection time *t* and the injection rate *v* of the cluster solution. Additionally, the first absorption maximum of the resulting QDs is provided. Optional annealing, with a specific reaction time *t*, was applied, and the resulting first absorption maxima are listed. Precipitation techniques were used to isolate the resulting QDs. The concentrations and sizes of the seeds and clusters were not determined by direct weighing but were standardized using absorption spectroscopy (OD at the absorption maximum).

#	InAs seeds			InAs clusters			*t* (min)	*v* (mL h^{-1})	1st abs. peak of QDs (nm)	+ annealing	
	λ (nm)	OD	*V* (mL)	*λ* (nm)	OD	*V* (mL)				*t* (min)	1st abs. peak (nm)
1	1,054	0.20	6.0	505	0.24	6.0	120	3	1,450	-	-
2	1,054	0.20	6.0	489	0.10	16.0	27	36	1,504	-	-
3	910	0.38	6.0	497	0.22	6.0	30	12	-	30	1,260

Supplementary Table 4: Concentrations of the InAs cluster and seed solutions, derived from the absorption spectra in Suppl. Fig. 18 (OD and wavelength) for the standard seeded growth synthesis of large InAs QDs.[1,2] The OD at the target first absorption peak of each solution was adjusted by dilution with dry OAm (see Methods).

solution	OD	wavelength
standard InAs cluster solution	0.22	526 nm
standard InAs seed solution	0.16	931 nm

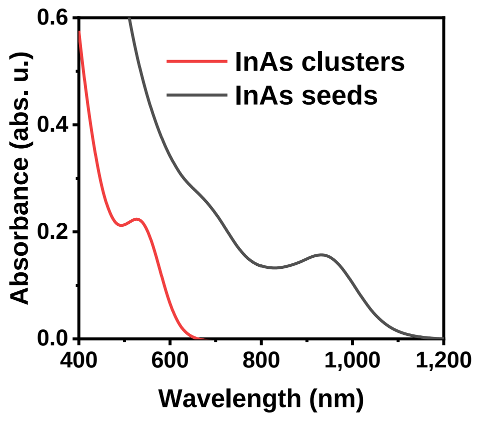

Supplementary Fig. 18: Absorption spectra of InAs cluster solution (red) and InAs seed solution (black), used for the standard seeded growth synthesis of large InAs QDs, corresponding to Suppl. Table 4.[1,2] Source data are provided as a Source Data file.

Supplementary Table 5: Overview of the resulting first absorption maxima, average particle sizes, and theoretical absorption maxima (calculated from Equation 1 in ref.[4]) from the average particle sizes of InAs QD samples synthesized via the standard seeded growth protocol, depending on the number of cluster injection and annealing steps.[1,2] Size-selective precipitation techniques were applied.

step #	standard seeded growth procedure	1st absorption peak of the fraction with the largest InAs QDs	average particle sizes of the fraction with the largest InAs QDs	theoretical 1st absorption peak, calculated according to Equation 1 from ref.[4], for average QD sizes determined from TEM images
1	1st injection of 4.5 mL of clusters	1,151 nm	5.7 ± 0.6 nm	1,136 nm
2	1st annealing 30 min, 300 °C	1,211 mn	6.2 ± 1.0 nm	1,201 nm
3	2nd injection of 4.5 mL of clusters	1,462 nm	8.5 ± 1.0 nm	1,471 nm
4	2nd annealing 30 min, 300 °C	1,530 nm	9.2 ± 0.9 nm	1,544 nm
5	3rd injection of 4.5 mL of clusters	1,605 nm	10.0 ± 1.5 nm	1,623 nm
6	3rd annealing 30 min, 300 °C	1,673 nm	10.5 ± 1.4 nm	1,671 nm
7	4th injection of 4.5 mL of clusters	1,771 nm	11.8 ± 1.3 nm	1,787 nm
8	4th annealing 30 min, 300 °C	≈1,800 nm	12.3 ± 2.0 nm	1,829 nm
9	5th injection of 4.5 mL of clusters	1,864 nm	12.9 ± 1.8 nm	1,876 nm
10	5th annealing 30 min, 300 °C	≈1,940 nm	13.8 ± 2.0 nm	1,943 nm
11	6th injection of 4.5 mL of clusters	≈1,990 nm	14.6 ± 2.9 nm	2,003 nm
16	8th annealing 30 min, 300 °C	≈2,300 nm	18.1 ± 3.3 nm	2,226 nm

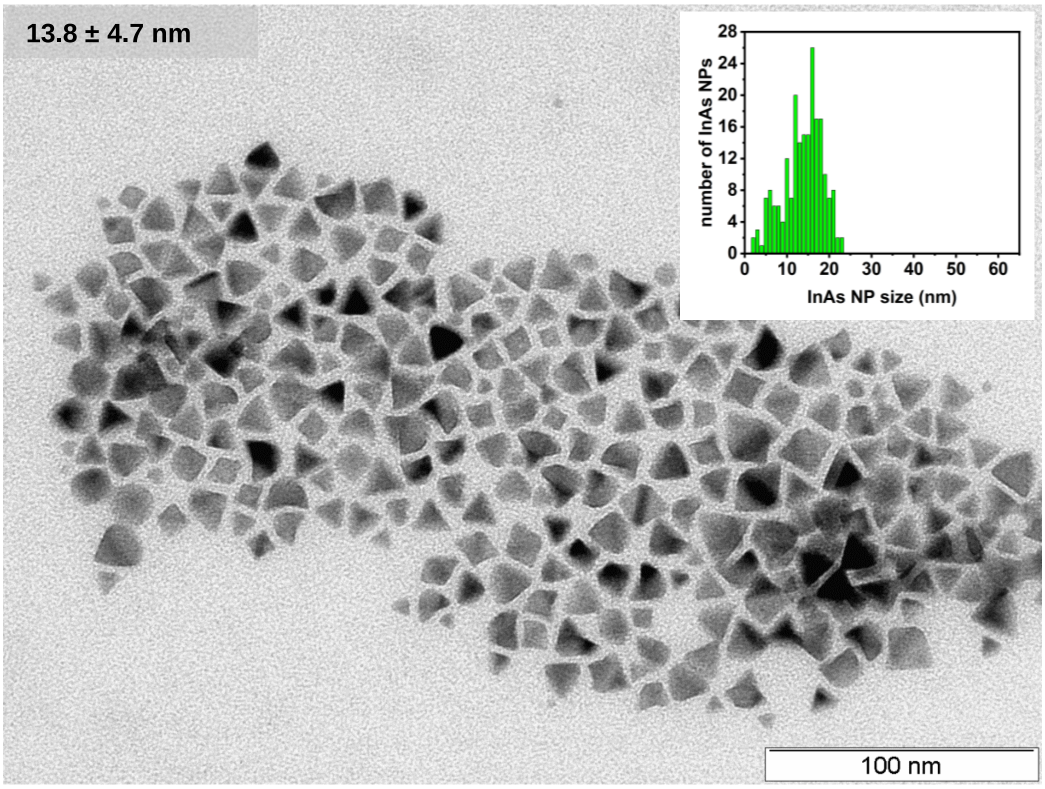

Supplementary Fig. 19: Purified InAs QDs, including a size distribution histogram, obtained in a 16-step standard seeded growth synthesis. No size-selective precipitation techniques were applied.

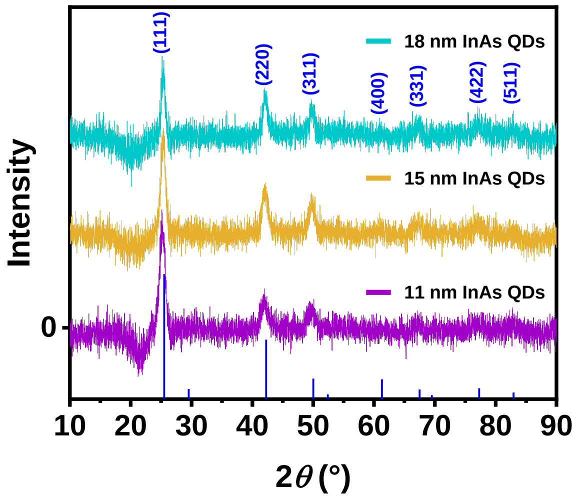

Supplementary Fig. 20: XRD diffractograms of large InAs QDs of different sizes, synthesized via the seeded growth method, are shown with offset and align with the bulk phase cubic crystal structure of InAs.[1,2] The samples were covered with Kapton® polyimide tape, and the background of an empty, tape-covered wafer was subtracted from the measured data. Source data are provided as a Source Data file.

Supplementary Table 6: Overview of the first absorption maxima of InAs QD samples synthesized via the seeded growth control method without annealing steps, depending on the volume of continuously injected InAs cluster solution.[1,2] Size-selective precipitation techniques were applied.

step #	seeded growth procedure (without annealing steps)	1st absorption peak of the fraction with largest InAs QDs
1	1st injection of 4.5 mL of clusters	1,131 nm
2	2nd injection of 4.5 mL of clusters	1,397 nm
3	3rd injection of 4.5 mL of clusters	1,513 nm
4	4th injection of 4.5 mL of clusters	1,565 nm
5	5th injection of 4.5 mL of clusters	1,750 nm

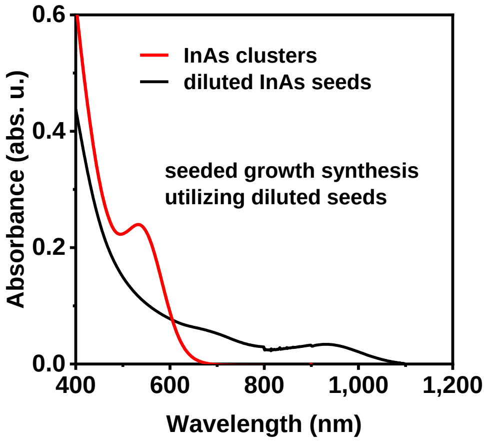

Supplementary Fig. 21: Absorption spectra of InAs cluster solution (red) and diluted InAs seed solution (black), corresponding to Suppl. Tab. 7, used for the seeded growth synthesis utilizing diluted seeds.[1,2] Source data are provided as a Source Data file.

Supplementary Table 7: InAs cluster and InAs seed solution concentration derived from the absorption spectra in Suppl. Fig. 12 (OD and wavelength) for the seeded growth synthesis of large InAs QDs utilizing diluted seeds.[1,2] The OD at the target first absorption peak of each solution was adjusted by dilution with dry OAm (see Methods).

solution	OD	wavelength
standard InAs cluster solution	0.23	532 nm
diluted InAs seed solution	0.034	931 nm

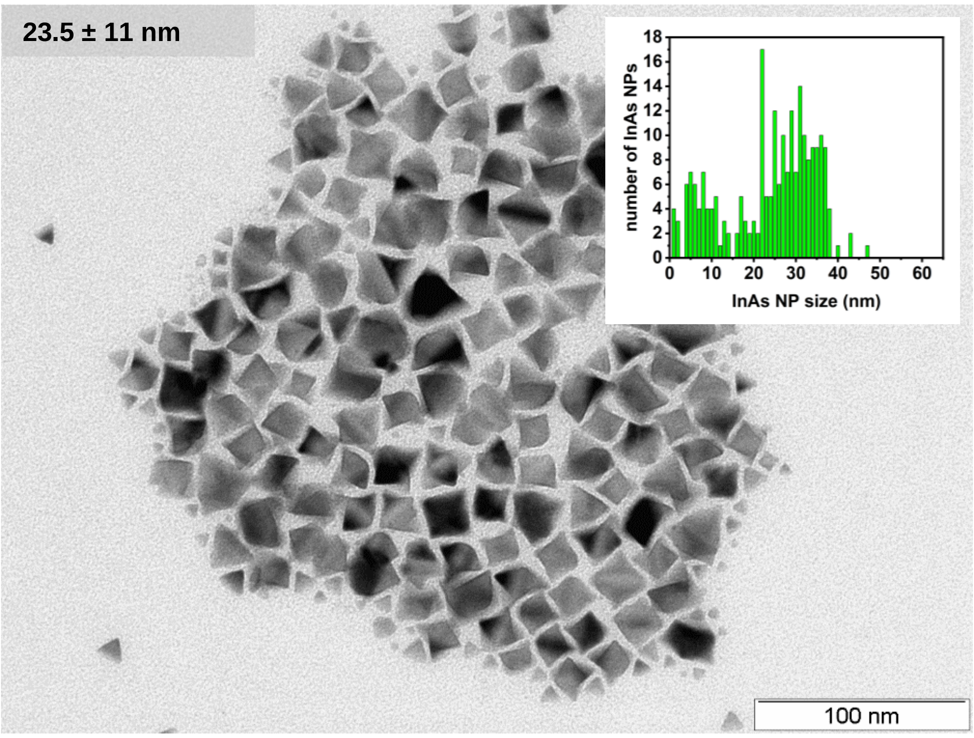

Supplementary Fig. 22: Purified InAs QDs, including a size distribution histogram, obtained in a 10-step seeded growth synthesis utilizing diluted seeds. No size-selective precipitation techniques were applied.

Supplementary Table 8: Overview of the average InAs QD sizes of samples synthesized via the seedless growth method, depending on the number of cluster injection and annealing steps.[1,2] Size-selective precipitation techniques were applied (see Methods).

step #	procedure	average InAs QD sizes in the fraction with largest InAs QDs (1st absorption maxima if possible)
1	1st injection of 4.5 mL of clusters	13.8 ± 2.0 nm (≈ 1,684 nm)
2	1st annealing 30 min, 300 °C	14.1 ± 1.9 nm (≈ 1,703 nm)
3	2nd injection of 4.5 mL of clusters	17.4 ± 2.2 nm
4	2nd annealing 30 min, 300 °C	18.7 ± 2.5 nm
5	3rd injection of 4.5 mL of clusters	22.5 ± 2.9 nm
6	3rd annealing 30 min, 300 °C	23.2 ± 3.6 nm
7	4th injection of 4.5 mL of clusters	28.3 ± 5.2 nm
8	4th annealing 30 min, 300 °C	30.9 ± 6.2 nm
9	5th injection of 4.5 mL of clusters	37.2 ± 4.4 nm
10	5th annealing 30 min, 300 °C	40.5 ± 7.5 nm

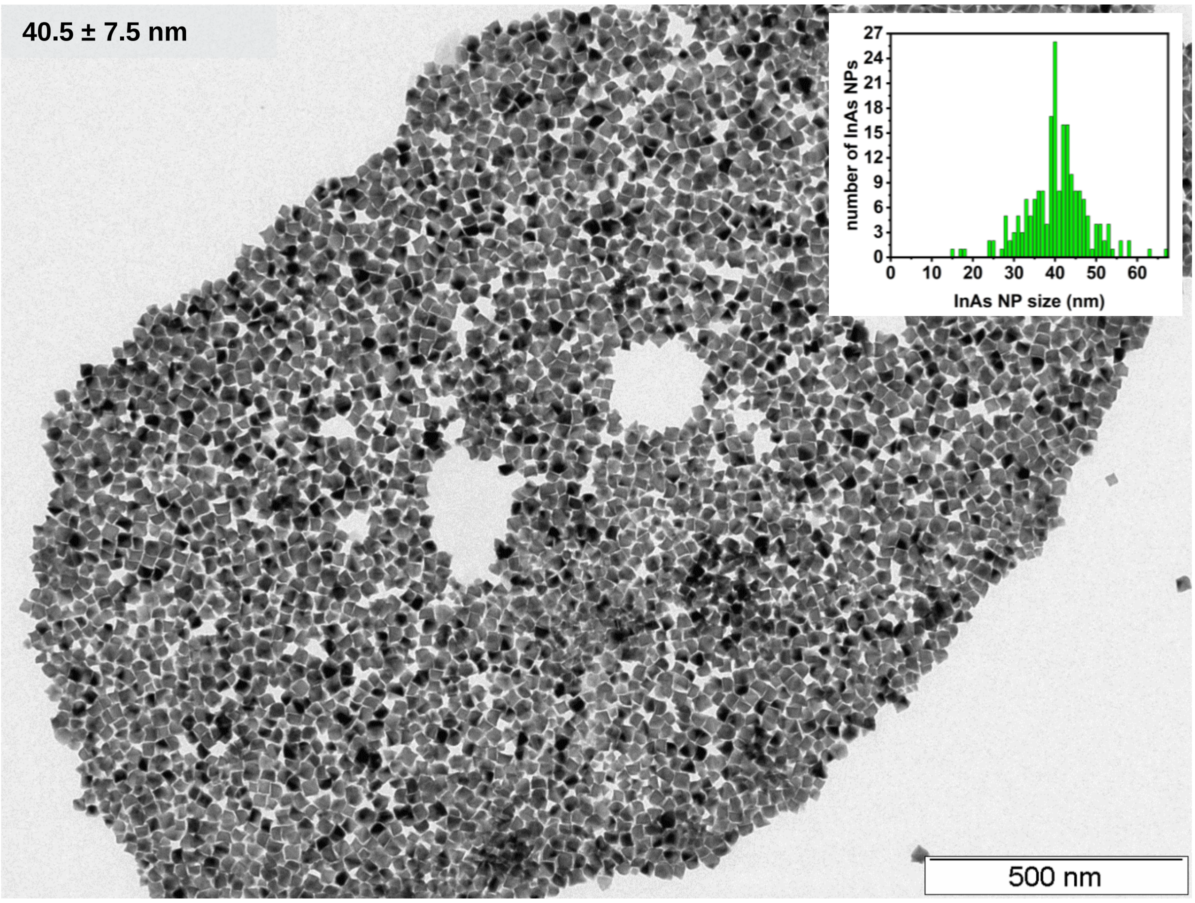

Supplementary Fig. 23: TEM image and size distribution histogram of large InAs NPs with an average diameter of 40 nm, synthesized via the 10-step seedless growth method.[1,2] Size-selective precipitation techniques were applied. Several areas exhibit favorable stacking behavior.

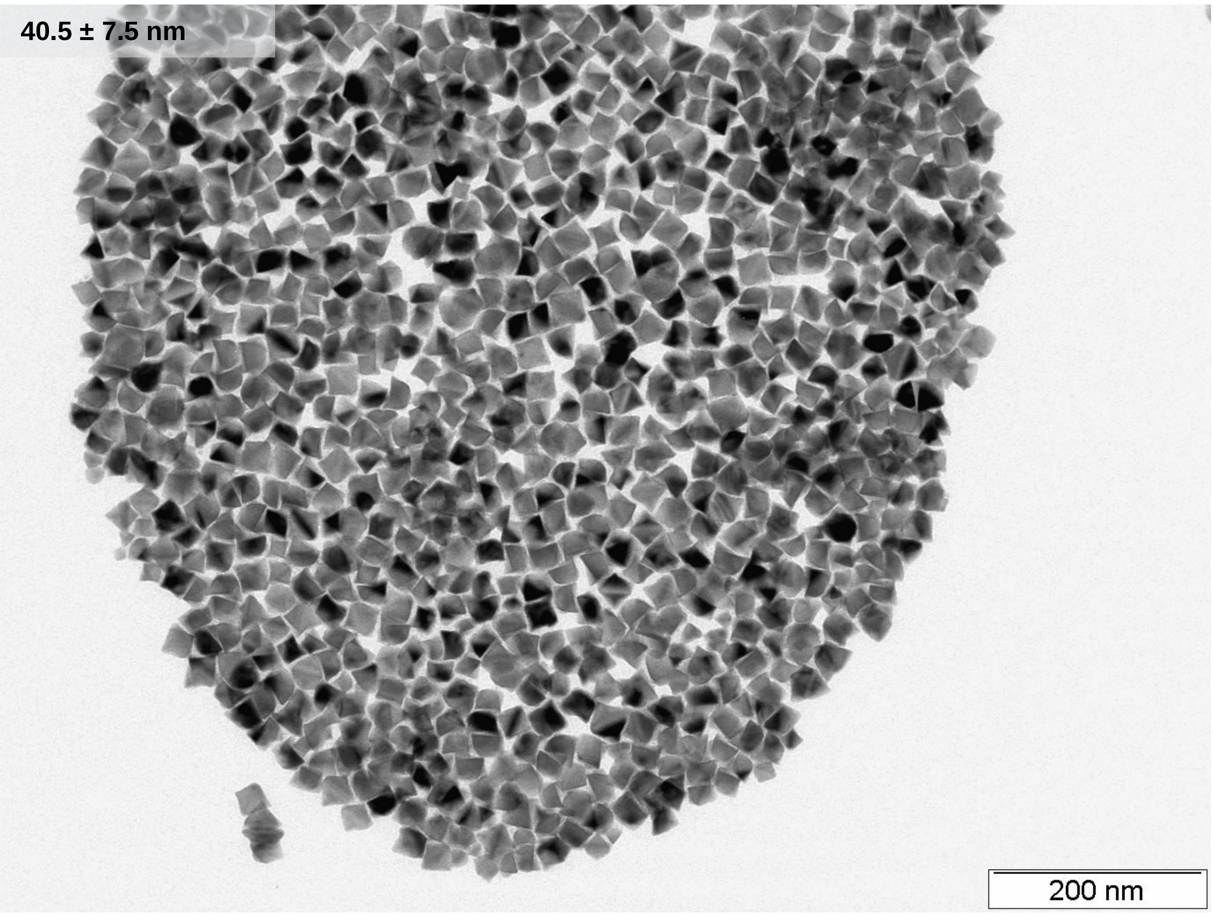

Supplementary Fig. 24: Additional large-scale TEM image of large InAs NPs with an average diameter of 40 nm, synthesized via the 10-step seedless growth approach.[2] Size-selective precipitation techniques were applied.

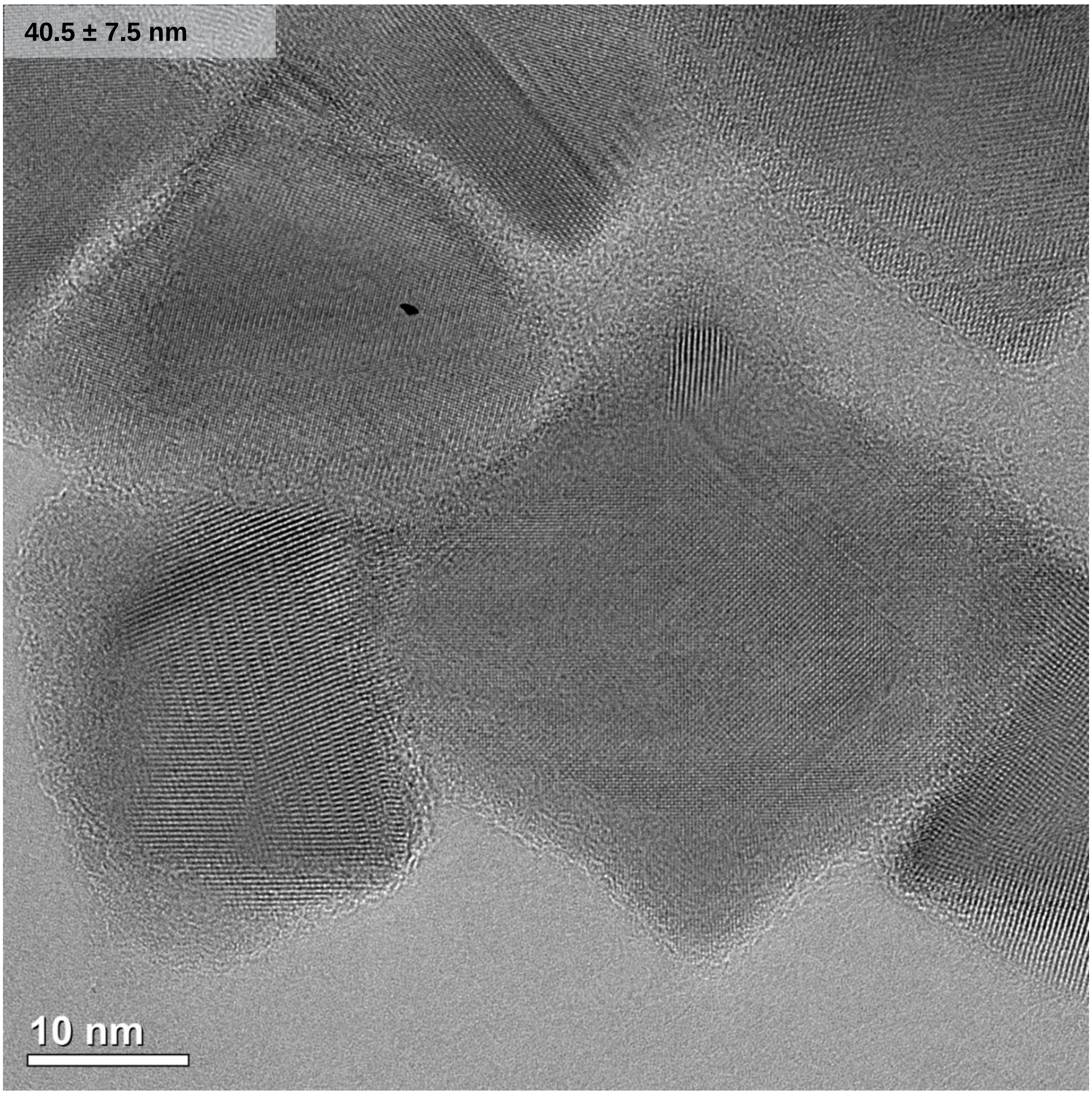

Supplementary Fig. 25: HR-TEM image of large InAs NPs synthesized via the seedless growth method.[1,2] Size-selective precipitation techniques were applied.

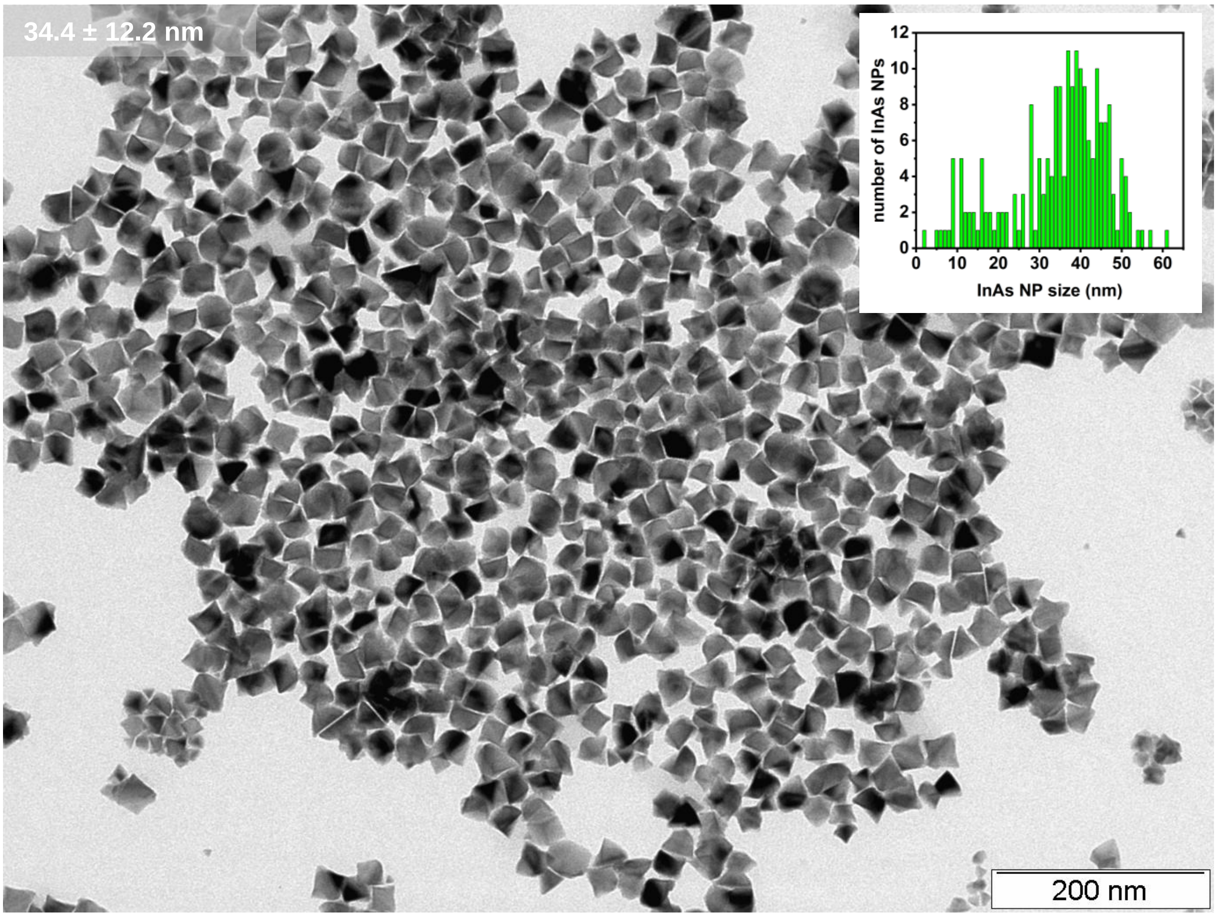

Supplementary Fig. 26: Purified InAs NPs, including a size distribution histogram, obtained in a 10-step seedless growth synthesis. No size-selective precipitation techniques were applied.

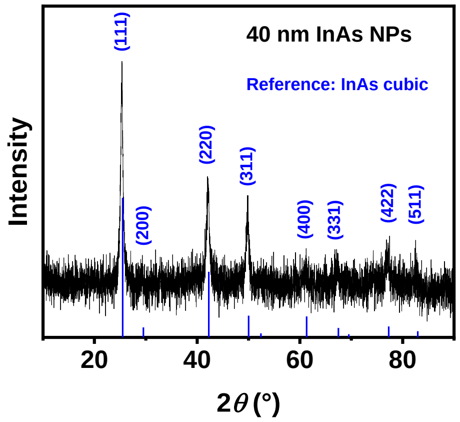

Supplementary Fig. 27: XRD diffractogram of large InAs NPs synthesized via the seedless growth method, confirming accordance with the bulk phase cubic crystal structure of InAs.[1,2] Source data are provided as a Source Data file.

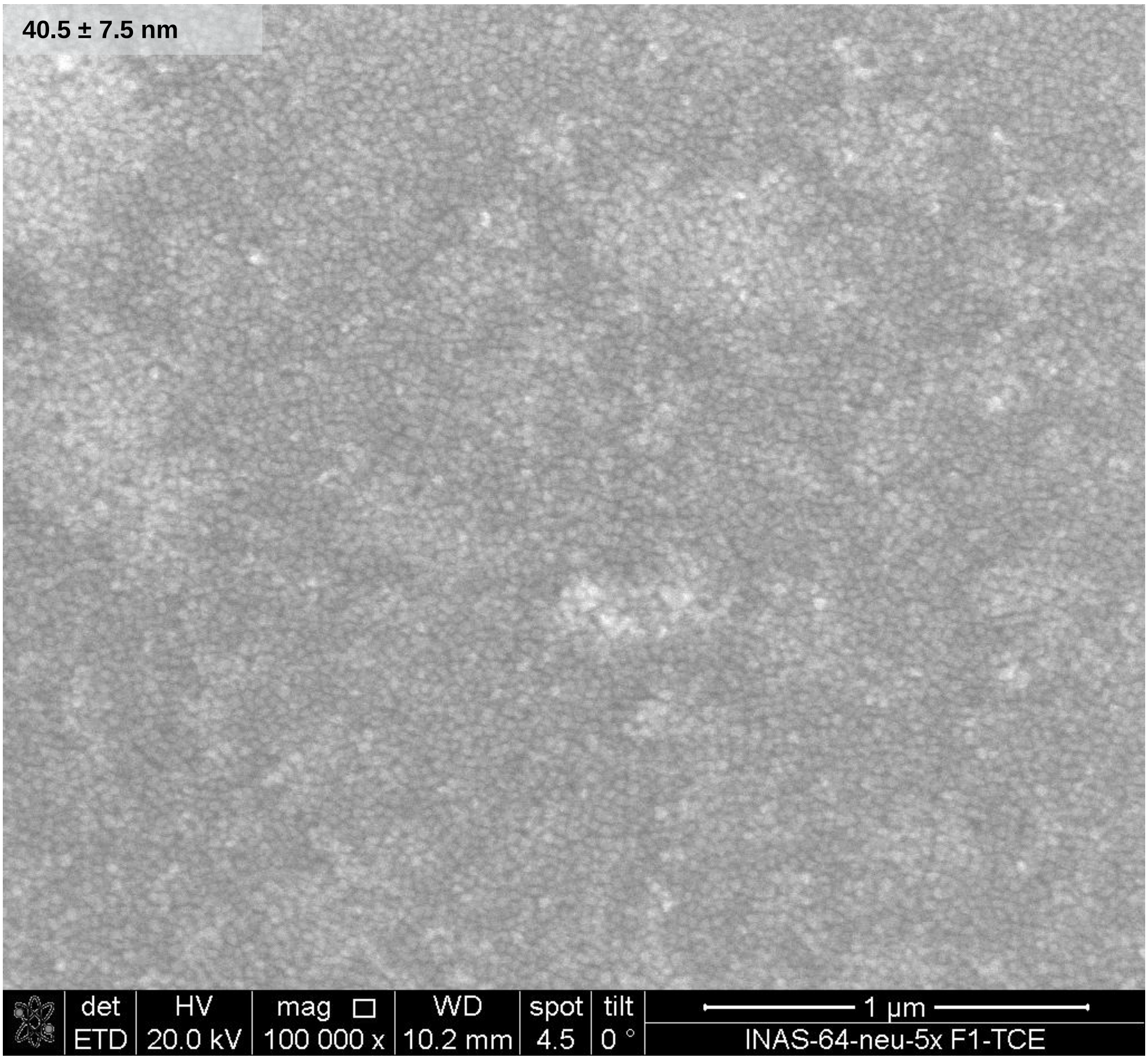

Supplementary Fig. 28: SEM image of the dried film produced via drop-casting of large InAs NPs; size-selective precipitation techniques were applied.[1,2] It reveals favorable stacking behavior of the particles, positioning them as promising candidates for layer-processing applications such as photovoltaics, quantum light emitting diodes (QLEDs), and sensors.

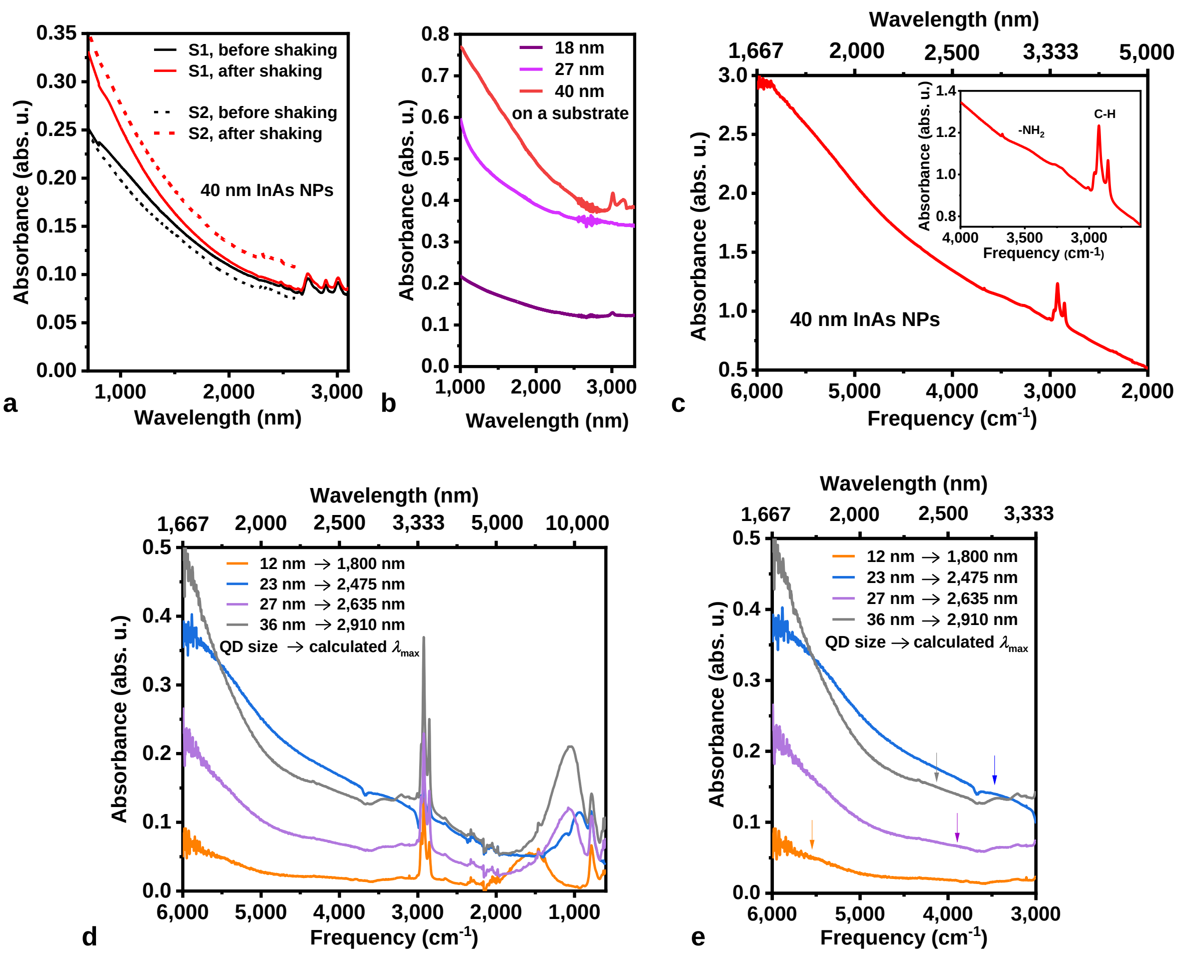

Supplementary Fig. 29: Spectroscopy on large InAs NPs synthesized via the seedless growth approach. Size-selective precipitation techniques were applied.[1,2] Source data are provided as a Source Data file. **a** Absorption spectra of two InAs NP suspensions with different concentrations (sample 1, S1, and sample 2, S2) in TCE, recorded before (black curves) and after (red curves) shaking.[1,2] Absorption bands of OAm ligands above 2,600 nm are present.[1,2] **b** Absorption spectra of InAs NPs measured on a substrate. **c** ATR-IR spectrum of a thin film of 40 nm large InAs NPs, including a zoomed-in inset, and **d** ATR-IR spectra and **e** the zoomed-in spectra of InAs NPs with sizes between 12 – 36 nm. The NPs were capped with native OAm and TOP ligands. Arrows in **e** indicate the positions of the first absorption maxima calculated using the formula (Equation 1) from ref.[4]

Supplementary Note 2: During the first step of the seedless growth synthesis, by continuous injection of 4.5 mL of the deep red cluster solution (1.1 mg mL^{-1}) in OAm-TOP into 6.0 mL of the hot OAm within 45 min (6 mL h^{-1}), the solution initially appeared slightly yellowish (see Suppl. Fig. 30b-c) attributable to the color of pure OAm solvent (Suppl. Fig. 30a), without any noticeable color change for the first ≈2.3 mL of injected cluster solution. Subsequently, with continued cluster injection, the color rapidly shifted to dark yellow, then brownish, and finally dark brown (Suppl. Fig. 30d-j). No red color from cluster solution could be seen during the whole cluster injection process, indicating the rapid cluster transformation process.[1,2]

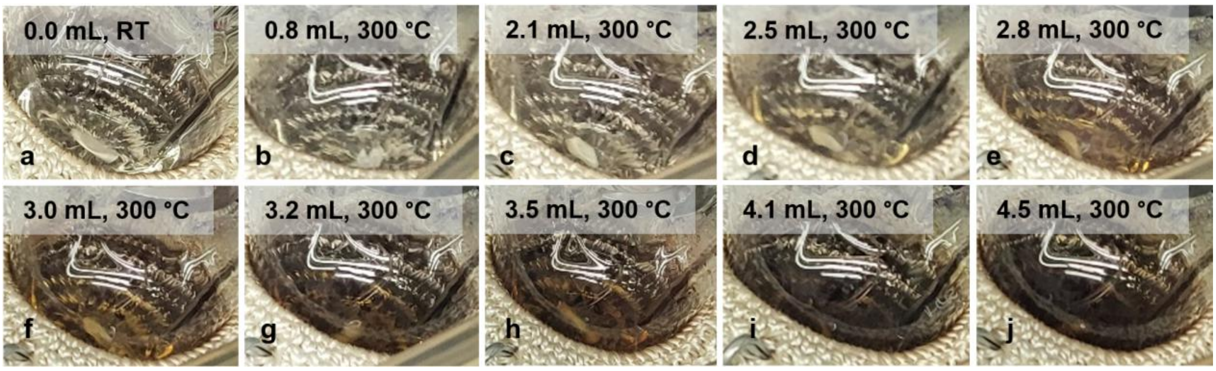

Supplementary Fig. 30: Photographs of the reaction mixture taken during the first step of the seedless growth synthesis.[1,2] **a** Pure OAm at RT **b-j** Reaction mixture at 300 °C during continuous injection of the InAs cluster solution in OAm-TOP, with the injected volume (in mL) indicated.

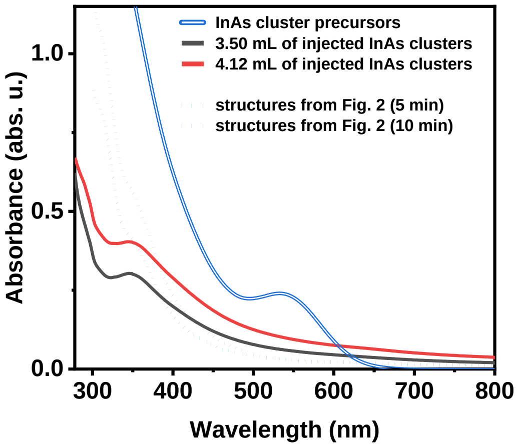

Supplementary Fig. 31: Absorption spectra of InAs cluster precursor solution (blue double line) and unpurified aliquots taken during the seedless growth synthesis, corresponding to Suppl. Fig. 30 (solid black and red lines), depending on the injected cluster volume. Also shown are representative early-stage structures from Fig. 3c (dashed green and violet lines).[1,2] Source data are provided as a Source Data file.

Supplementary Note 3: The reaction yield is estimated based on the weight of the inorganic products, as determined by thermogravimetric analysis (TGA) and discussed in Methods. The reaction yield of the InAs cluster synthesis, conducted at 130 °C for 76 min, was calculated as follows.

The reaction yield n according to In (from InCl, 0.300 g, 1.996 mmol) was calculated using Suppl. Equation 1:

$$n = \frac{0.316 \text{ mmol}}{1.996 \text{ mmol}} \triangleq 16\% \quad (1)$$

The reaction yield n according to As (from amino-As, 121.44 mg, 0.64 mmol) was calculated in Suppl. Equation 2:

$$n = \frac{0.316 \text{ mmol}}{0.64 \text{ mmol}} \triangleq 49\% \quad (2)$$

(2)

Concentration of the InAs cluster stock solution after separation of insoluble byproducts (Suppl. Equation 3):

$$c = \frac{5.7\ \mathrm{mg}}{3.0\ \mathrm{mL}} = 1.9\ \mathrm{mg\ mL^{-1}} \tag{3}$$

The concentration and mass of InAs clusters in 4.5 mL solution are listed in Suppl. Table 9.

Approximately 100% - 16% = 84% of the initially used InCl is converted into insoluble byproducts during the cluster synthesis. The high percentage is attributed to the disproportionation reaction of InCl,[5] which also explains the brown-grey color of the cluster mixture prior to centrifugation or sedimentation. The insoluble byproducts were primarily metallic In, with smaller amounts of In_2O_3 and InAs, as observed in the XRD analysis (see Suppl. Fig. 1).

Supplementary Table 9: Concentration and mass of InAs cluster precursors within 4.5 mL solution after removing insoluble byproducts.

solution	concentration	OD at absorption maximum	InAs mass from TGA in 4.5 mL solution
stock solution	1.9 mg mL^{-1}	0.39	8.6 mg (45 µmol)
cluster precursor solution	1.1 mg mL^{-1}	0.22	4.9 mg (26 µmol)

Supplementary Note 4: To determine the reaction yield of the InAs seed synthesis, procedure described in Methods was used.

The reaction yield n according to In (from InCl, 0.285 g, 1.897 mmol) was calculated using Suppl. Equation 4:

$$n = \frac{0.43\ \mathrm{mmol}}{1.897\ \mathrm{mmol}} \triangleq 23\% \tag{4}$$

The reaction yield n according to As (from amino-As, 0.09107 g, 0.48 mmol) was calculated in Suppl. Equation 5:

$$n = \frac{0.43\ \mathrm{mmol}}{0.48\ \mathrm{mmol}} \triangleq 90\% \tag{5}$$

Suppl. Tab. 10 shows the seed concentrations calculated using TGA, the OD at the absorption maximum, and the InAs mass in 6.0 mL solution for three different concentrated seed solutions: the stock solution, the solution used for the standard seeded growth method, and solution used for the diluted seeds approach. The OD values are also listed.

Supplementary Table 10: Concentration and mass of InAs seeds within 6.0 mL solution after removing insoluble byproducts.

solution	concentration	OD at absorption maximum	InAs mass from TGA in 6.0 mL solution
stock solution	3.3 mg mL^{-1}	0.46	20.0 mg (105 µmol)
standard seeded growth	1.3 mg mL^{-1}	0.16	8.0 mg (42 µmol)
diluted seeds approach	0.24 mg mL^{-1}	0.034	1.4 mg (7.5 µmol)

Supplementary Note 5: The reaction yield of the 16-step standard seeded growth synthesis was determined as described in Methods.

The theoretical mass of InAs QDs calculated from the mass of seeds (8.0 mg) and clusters, added 8 times 4.5 mL (containing 4.9 mg of InAs) in the 16-step standard seeded growth approach (Suppl. Equation 6):

$$m_{\text{seeds}} + m_{\text{clusters}} = 8.0\ \text{mg} + 4.9\ \text{mg} * 8 = 47.2\ \text{mg} \tag{6}$$

The reaction yield n according to InAs synthesized using clusters and seeds (47.2 mg, 0.249 mmol) was calculated using Suppl. Equation 7:

$$n = \frac{0.19\ \text{mmol}}{0.249\ \text{mmol}} \triangleq 76\% \tag{7}$$

Supplementary Note 6: The reaction yield of the **10-step seedless growth synthesis** was determined as described in Methods.

The theoretical mass of InAs QDs calculated from the mass of clusters, added 5 times 4.5 mL (containing 4.9 mg of InAs) in the 10-step seedless growth approach (Suppl. Equation 8):

$$m_{\text{clusters}} = 4.9\ \text{mg} * 5 = 24.8\ \text{mg} \tag{8}$$

The reaction yield n according to InAs synthesized from clusters (24.8 mg, 0.131 mmol), calculated in Suppl. Equation 9:

$$n = \frac{0.129\ \text{mmol}}{0.131\ \text{mmol}} \triangleq 98\% \tag{9}$$

The reaction yield of the **6-step seedless growth synthesis** was determined as described in Methods.

The theoretical mass of InAs QDs calculated from the mass of clusters, added 3 times 4.5 mL (containing 4.9 mg of InAs) in the 6-step seedless growth approach (Suppl. Equation 10):

$$m_{\text{clusters}} = 4.9\ \text{mg} * 3 = 14.9\ \text{mg} \tag{10}$$

The reaction yield n according to InAs synthesized from clusters (14.9 mg, 0.0786 mmol), Suppl. Equation 11:

(11)

$$n = \frac{0.0896\ \mathrm{mg}}{0.0786\ \mathrm{mmol}} \triangleq 114\%$$

The value above 100% indicates deviations, likely caused by the low percentage of inorganic material (0.09%).

By comparing the mass of the resulting InAs QD samples obtained at different synthesis stages of the seedless growth synthesis (15 mg after 6 steps, 25 mg after 10 steps), it is evident that continued QD growth takes place, which is consistent with observations from TEM analysis and absorption measurements. Since the seedless growth synthesis begins without any InAs material in the flask, the overall reaction yield after the same number of steps is lower than that observed in the seeded growth process.

Supplementary References

[1] Salikhova, E., Mews, A. & Niehaus, Jan Steffen. Colloidal synthesis of ultra-large InAs quantum dots. *ChemRxiv*. https://doi.org/10.26434/chemrxiv-2025-hgkl3 (2025).

[2] Salikhova, E. Colloidal synthesis of InAs nanocrystals: from clusters to ultra-large quantum dots, InAs/ZnS quantum dots with improved photoluminescence, and luminescent `giant` shell InAs/ZnSe/ZnS quantum dots *via* `green` routes. Dissertation, *Universität Hamburg*, https://ediss.sub.uni-hamburg.de/handle/ediss/12035 (2025).

[3] Raghavendra, R., Arunchalam, K., Annamali, S. K. & Arunachalam, A. Diagnostics and therapeutic application of gold nanoparticles. *Int J Pharm Pharm Sci* 6, 2, 74-87 (2014).

[4] Kuno, M., Gushchina, I., Toso, S. & Trepalin, V. No one size fits all: semiconductor nanocrystal sizing curves. *J. Phys. Chem. C* 126, 11867−11874 (2022).

[5] Ginterseder, M. et al. Scalable synthesis of InAs quantum dots mediated through indium redox chemistry. *J. Am. Chem. Soc.* **142**, 4088−4092 (2020).
